# Crystal structure of (*E*)-*N*-[(2-chloro-6-meth­oxy­quinolin-3-yl)methyl­idene]-9-ethyl-9*H*-carbazol-3-amine

**DOI:** 10.1107/S2056989015005794

**Published:** 2015-03-28

**Authors:** Kannan Thirumurthy, Ganesamoorthy Thirunarayanan, S. Murugavel

**Affiliations:** a Department of Chemistry, Annamalai University, Annamalainagar 608 002, Chidambaram, Tamilnadu, India; b Department of Physics, Thanthai Periyar Government Institute of Technology, Vellore 632 002, India

**Keywords:** crystal structure, crystal packing, quinoline, carbazole, 9-ethyl-9*H*-carbazol-3-amine, C—H⋯π inter­actions, π–π inter­actions

## Abstract

In the title compound, C_25_H_20_ClN_3_O, the C=N bond of the central imine group adopts an *E* conformation. The mean planes through the essentially planar carbazole [maximum deviation = 0.052 (2)Å] and quinoline [maximum deviation = 0.050 (2) Å] ring systems form a dihedral angle of 50.2 (1)°. In the crystal, mol­ecules are linked by C—H⋯π and π—π inter­actions [centroid–centroid distances ranging from 3.635 (2) to 3.739 (2) Å], forming a three-dimensional supra­molecular network.

## Abstract 2

In the title compound, C_25_H_20_ClN_3_O, the dihedral between the carbazole and quinoline ring systems is 50.2 (1)°. The crystal packing features C—H⋯π and π—π inter­actions, which generate a three-dimensional network.

## Chemical context   

It has been reported that carbazole derivatives possess various biological activities, such as anti­tumor (Itoigawa *et al.*, 2000 ▸), anti-oxidative (Tachibana *et al.*, 2001 ▸), anti-inflammatory and anti­mutagenic (Ramsewak *et al.*, 1999 ▸). Carbazole derivatives also exhibit electroactivity and luminescence properties and are considered to be potential candidates for electronic devices such as colour displays, organic semiconductor lasers and solar cells (Friend *et al.*, 1999 ▸). These compounds are thermally and photochemically stable, which makes them useful materials for technological applications: for instance, the carbazole ring is easily funtionalized and covalently linked to other mol­ecules (Díaz *et al.*, 2002 ▸). This enables its use as a convenient building block for the design and synthesis of mol­ecular glasses, which are widely studied as components of electroactive and photoactive materials (Zhang *et al.*, 2004 ▸). Quinoline derivatives are known to possess a variety of biological properties such as anti­malarial and anti­viral activity (Cunico *et al.*, 2006 ▸; Hartline *et al.*, 2005 ▸). Against this background, and in order to obtain detailed information on its mol­ecular conformation in the solid state, the crystal structure of the title compound has been determined.
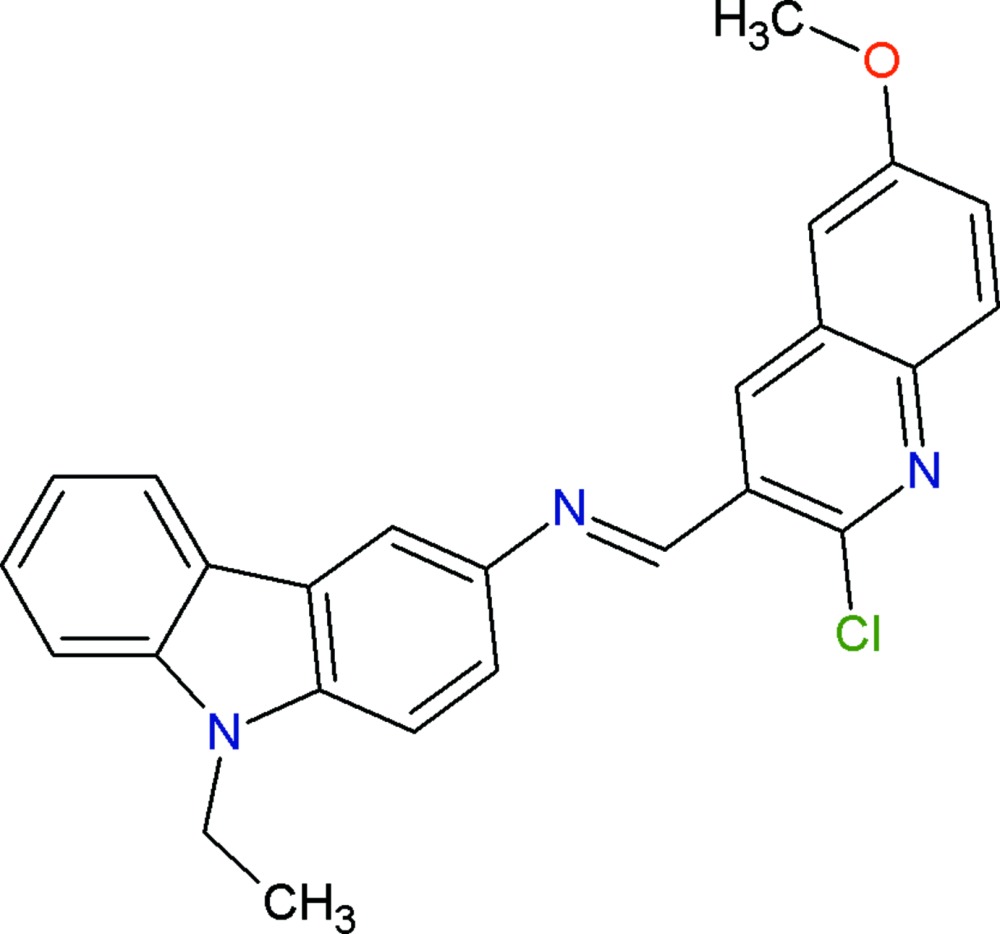


## Structural commentary   

**Figure 1:**
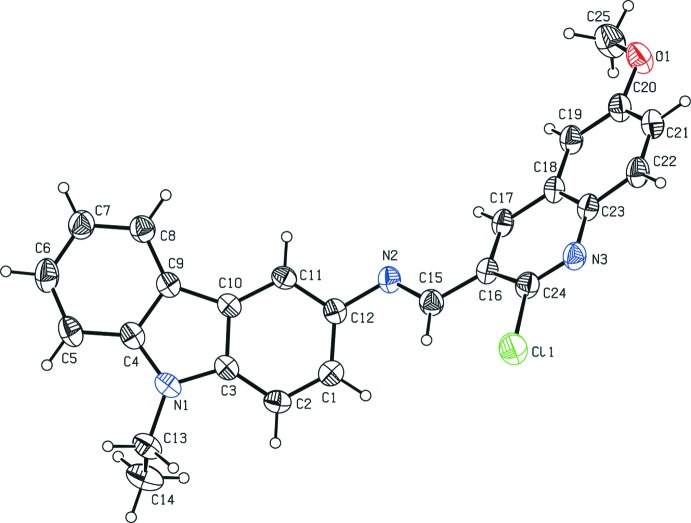
Mol­ecular structure of the title compound showing displacement ellipsoids at the 30% probability level. H atoms are drawn as a small spheres of arbitrary radii.

Fig. 1 ▸. shows a displacement ellipsoid plot of (I), with the atom-numbering scheme. The C=N bond of the central imine group adopts an *E* conformation. The mean planes through the essentially planar carbazole [N1/C1–C12; maximum deviation = 0.052 (2) Å for atom C12] and quinoline [N3/C16–C24; maximum deviation = 0.050 (2) Å for atom C16] ring systems form a dihedral angle of 50.2 (1)°. The sum of the bond angles around N1 (360.05°) of the pyrrole ring is in accordance with *sp* ^2^ hybridization. Atom Cl1 deviates from the plane of the attached quinoline ring system by 0.100 (1) Å. The geometric parameters of the title mol­ecule agree well with those reported for similar structures (Murugavel *et al.*, 2009 ▸; Archana *et al.*, 2011 ▸).

## Supra­molecular features   

**Figure 2:**
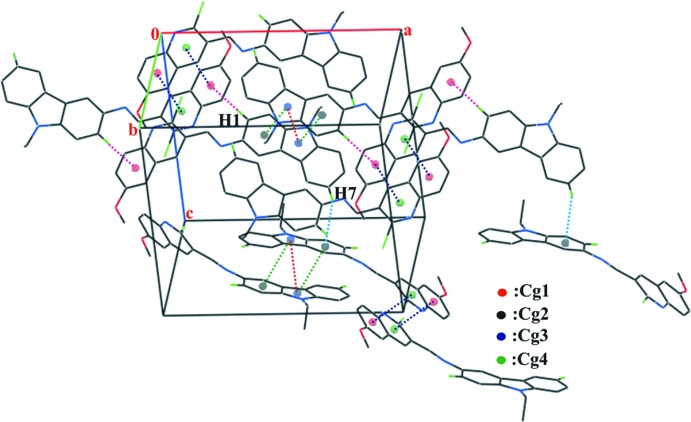
Part of the crystal structure of the title compound showing the C—H⋯π and π–π inter­actions, which lead to the formation of a three-dimensional supra­molecular network. Hydrogen atoms not involved in hydrogen bonding are omitted for clarity. *Cg*1, *Cg*2, *Cg*3 and *Cg*4 are the centroids of the C18–C23 benzene ring, the C1–C3/C10–C12 benzene ring, the N1/C3/C4/C9/C10 pyrrole ring and the N3/C16–C18/C23/C24 pyridine ring, respectively.

In the crystal, mol­ecules are linked by two C—H⋯π inter­actions; the first one between the benzene H atom of the carbazole ring system and the benzene ring of an adjacent mol­ecule, with a C1—H1⋯*Cg*1^i^ and the second one between the benzene H atom of the carbazole ring system and the benzene ring of an adjacent mol­ecule, with a C7—H7⋯*Cg*2^ii^. The mol­ecules are further linked by π–π inter­actions with *Cg*3–*Cg*3^iii^, *Cg*3–*Cg*2^iii^, *Cg*2–*Cg*3^iii^, *Cg*4–*Cg*1^iv^ and *Cg*1–*Cg*4^iv^ separations of 3.735 (2), 3.739 (2), 3.739 (2), 3.635 (2) and 3.635 (2) Å, respectively, forming a three-dimensional supra­molecular network (Table 1 ▸ and Fig. 2 ▸; *Cg*1, *Cg*2, *Cg*3 and *Cg*4 are the centroids of C18–C23 benzene ring, the C1–C3/C10–C12 benzene ring, the N1/C3/C4/C9/C10 pyrrole ring and the N3/C16–C18/C23/C24 pyridine ring, respectively; symmetry codes: (i) −*x*, −*y*, 1 − *z*; (ii) 1 − *x*, 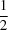 + *y*, 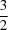 − *z*; (iii) 1 − *x*, −*y*, 1 − *z* and (iv) −*x*, 1 − *y*, 1 − *z*).

## Synthesis and crystallization   

A 25 ml round-bottom flask was charged with dimedone (1 mmol), 2-chloro-6-meth­oxy­quinoline-3-carbaldehyde (1 mmol) 9-ethyl-9*H*-carbazol-3-amine (1 mmol) and sulfated SnO_2_-fly ash catalyst (50 mg) in water (15 ml) and was refluxed at 353 K for 5–10 minutes. The completion of the reaction was monitored by TLC (ethyl acetate and hexane as an eluent 20%). After completion, the reaction mixture was cooled to ambient temperature. Then di­chloro­methane (20 ml) was added and the organic layer filtered, dried on anhydrous Na_2_SO_4_ and the solvent removed using a rotary evaporator. The crude product was purified by column chromatography on silica gel (200 mesh) with hexane and ethyl acetate (4:1) as eluent to afford the title compound in good yield (10%). Red blocks suitable for X-ray diffraction analysis were obtained by recrystallization from di­chloro­methane solution at room temperature.

## Refinement   

Crystal data, data collection and structure refinement details are summarized in Table 2 ▸. H atoms were positioned geom­etrically and constrained to ride on their parent atom with C—H = 0.93–0.97 Å and with *U*_iso_(H)=1.5*U*_eq_ for methyl H atoms and 1.2*U*_eq_(C) for other H atoms.

## Supplementary Material

Crystal structure: contains datablock(s) global, I. DOI: 10.1107/S2056989015005794/hb7384sup1.cif

Structure factors: contains datablock(s) I. DOI: 10.1107/S2056989015005794/hb7384Isup2.hkl

Supporting information file. DOI: 10.1107/S2056989015005794/hb7384Isup3.cml

CCDC reference: 1027676

Additional supporting information:  crystallographic information; 3D view; checkCIF report

## Figures and Tables

**Table 1:** Hydrogen-bond geometry (, ) *Cg*1 and *Cg*2 are the centroids of the C18C23 and C1C3/C10C12 benzene rings, respectively.

*D*H*A*	*D*H	H*A*	*D* *A*	*D*H*A*
C1H1*Cg*1^i^	0.93	2.87	3.718(3)	152
C7H7*Cg*2^ii^	0.93	2.97	3.688(2)	145

Symmetry codes: (i) 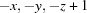; (ii) 
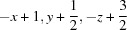
.

**Table 2:** Experimental details

Crystal data	
Chemical formula	C_25_H_20_ClN_3_O
*M*_r_	413.89
Crystal system, space group	Monoclinic, *P*2_1_/*c*
Temperature (K)	293
*a*, *b*, *c* ()	15.060(3), 8.8231(15), 15.332(3)
()	93.344(3)
*V* (^3^)	2033.9(6)
*Z*	4
Radiation type	Mo *K*
(mm^1^)	0.21
Crystal size (mm)	0.24 0.21 0.16

Data collection	
Diffractometer	Bruker SMART CCD
Absorption correction	Multi-scan (*SADABS*; Sheldrick, 1996 ▸)
*T*_min_, *T*_max_	0.951, 0.967
No. of measured, independent and observed [*I* > 2(*I*)] reflections	20538, 4023, 2340
*R*_int_	0.056
(sin /)_max_ (^1^)	0.620

Refinement	
*R*[*F* ^2^ > 2(*F* ^2^)], *wR*(*F* ^2^), *S*	0.051, 0.133, 1.01
No. of reflections	4023
No. of parameters	273
H-atom treatment	H-atom parameters constrained
_max_, _min_ (e ^3^)	0.24, 0.14

Computer programs: *SMART* and *SAINT* (Bruker, 2002 ▸), *SHELXS97* and *SHELXL97* (Sheldrick, 2008 ▸), *ORTEP-3 for Windows* (Farrugia (1997 ▸) and *PLATON* (Spek, 2009 ▸).

## supplementary crystallographic information

### Crystal data

**Table:**

C_25_H_20_ClN_3_O	*F*(000) = 864
*M**_r_* = 413.89	*D*_x_ = 1.352 Mg m^−^^3^
Monoclinic, *P*2_1_/*c*	Mo *K*α radiation, λ = 0.71073 Å
Hall symbol: -P 2ybc	Cell parameters from 4051 reflections
*a* = 15.060 (3) Å	θ = 1.4–26.1°
*b* = 8.8231 (15) Å	µ = 0.21 mm^−^^1^
*c* = 15.332 (3) Å	*T* = 293 K
β = 93.344 (3)°	Block, red
*V* = 2033.9 (6) Å^3^	0.24 × 0.21 × 0.16 mm
*Z* = 4	

### Data collection

**Table:**

Bruker SMART CCD diffractometer	4023 independent reflections
Radiation source: fine-focus sealed tube	2340 reflections with *I* > 2σ(*I*)
Graphite monochromator	*R*_int_ = 0.056
ω scans	θ_max_ = 26.1°, θ_min_ = 1.4°
Absorption correction: multi-scan (*SADABS*; Sheldrick, 1996)	*h* = −18→18
*T*_min_ = 0.951, *T*_max_ = 0.967	*k* = −10→10
20538 measured reflections	*l* = −18→18

### Refinement

**Table:**

Refinement on *F*^2^	Primary atom site location: structure-invariant direct methods
Least-squares matrix: full	Secondary atom site location: difference Fourier map
*R*[*F*^2^ > 2σ(*F*^2^)] = 0.051	Hydrogen site location: inferred from neighbouring sites
*wR*(*F*^2^) = 0.133	H-atom parameters constrained
*S* = 1.01	*w* = 1/[σ^2^(*F*_o_^2^) + (0.0565*P*)^2^ + 0.145*P*] where *P* = (*F*_o_^2^ + 2*F*_c_^2^)/3
4023 reflections	(Δ/σ)_max_ < 0.001
273 parameters	Δρ_max_ = 0.24 e Å^−^^3^
0 restraints	Δρ_min_ = −0.14 e Å^−^^3^

### Special details

**Table:**

Geometry. All esds (except the esd in the dihedral angle between two l.s. planes) are estimated using the full covariance matrix. The cell esds are taken into account individually in the estimation of esds in distances, angles and torsion angles; correlations between esds in cell parameters are only used when they are defined by crystal symmetry. An approximate (isotropic) treatment of cell esds is used for estimating esds involving l.s. planes.
Refinement. Refinement of F^2^ against ALL reflections. The weighted R-factor wR and goodness of fit S are based on F^2^, conventional R-factors R are based on F, with F set to zero for negative F^2^. The threshold expression of F^2^ > 2sigma(F^2^) is used only for calculating R-factors(gt) etc. and is not relevant to the choice of reflections for refinement. R-factors based on F^2^ are statistically about twice as large as those based on F, and R- factors based on ALL data will be even larger.

### Fractional atomic coordinates and isotropic or equivalent isotropic displacement parameters (Å^2^)

**Table:**

	*x*	*y*	*z*	*U*_iso_*/*U*_eq_	
Cl1	0.06066 (5)	0.06058 (9)	0.30880 (4)	0.0824 (3)	
C10	0.43767 (14)	0.0240 (2)	0.62074 (13)	0.0455 (6)	
C11	0.35381 (15)	0.0892 (3)	0.61516 (14)	0.0481 (6)	
H11	0.3441	0.1814	0.6424	0.058*	
N2	0.20216 (12)	0.0956 (2)	0.55982 (12)	0.0541 (5)	
O1	−0.20036 (11)	0.5826 (2)	0.63150 (12)	0.0760 (6)	
C23	−0.08229 (15)	0.3293 (3)	0.44161 (15)	0.0531 (6)	
N3	−0.04814 (13)	0.2485 (2)	0.37581 (12)	0.0585 (5)	
N1	0.53829 (13)	−0.1591 (2)	0.59420 (13)	0.0562 (5)	
C9	0.52392 (15)	0.0703 (3)	0.65834 (14)	0.0484 (6)	
C3	0.45006 (15)	−0.1178 (3)	0.58178 (15)	0.0491 (6)	
C12	0.28459 (15)	0.0167 (3)	0.56899 (14)	0.0497 (6)	
C4	0.58357 (16)	−0.0465 (3)	0.64053 (15)	0.0524 (6)	
C2	0.38021 (16)	−0.1946 (3)	0.53925 (15)	0.0566 (6)	
H2	0.3885	−0.2902	0.5155	0.068*	
C24	0.02130 (16)	0.1662 (3)	0.39528 (15)	0.0555 (6)	
C18	−0.04726 (14)	0.3209 (3)	0.52873 (15)	0.0494 (6)	
C19	−0.08694 (15)	0.4038 (3)	0.59474 (15)	0.0576 (6)	
H19	−0.0646	0.3964	0.6524	0.069*	
C16	0.06697 (14)	0.1538 (3)	0.47843 (15)	0.0509 (6)	
C20	−0.15802 (16)	0.4946 (3)	0.57397 (16)	0.0583 (7)	
C1	0.29845 (16)	−0.1263 (3)	0.53286 (15)	0.0569 (6)	
H1	0.2510	−0.1762	0.5038	0.068*	
C22	−0.15569 (16)	0.4262 (3)	0.42313 (17)	0.0634 (7)	
H22	−0.1796	0.4345	0.3660	0.076*	
C8	0.55487 (16)	0.1987 (3)	0.70283 (15)	0.0590 (7)	
H8	0.5159	0.2760	0.7161	0.071*	
C17	0.02964 (14)	0.2319 (3)	0.54418 (15)	0.0534 (6)	
H17	0.0558	0.2260	0.6006	0.064*	
C15	0.15054 (15)	0.0715 (3)	0.49281 (16)	0.0566 (6)	
H15	0.1664	−0.0001	0.4520	0.068*	
C6	0.70181 (18)	0.0939 (4)	0.70840 (17)	0.0737 (8)	
H6	0.7618	0.1043	0.7252	0.088*	
C13	0.57753 (17)	−0.2968 (3)	0.56101 (16)	0.0649 (7)	
H13A	0.6399	−0.2785	0.5522	0.078*	
H13B	0.5483	−0.3215	0.5048	0.078*	
C21	−0.19161 (16)	0.5068 (3)	0.48695 (17)	0.0635 (7)	
H21	−0.2392	0.5712	0.4731	0.076*	
C5	0.67368 (17)	−0.0363 (3)	0.66605 (16)	0.0645 (7)	
H5	0.7131	−0.1142	0.6549	0.077*	
C7	0.64419 (17)	0.2099 (3)	0.72704 (17)	0.0691 (7)	
H7	0.6658	0.2960	0.7561	0.083*	
C25	−0.17246 (19)	0.5688 (4)	0.72159 (18)	0.0863 (9)	
H25A	−0.1805	0.4660	0.7402	0.129*	
H25B	−0.2073	0.6352	0.7555	0.129*	
H25C	−0.1108	0.5957	0.7297	0.129*	
C14	0.5698 (2)	−0.4287 (3)	0.62109 (19)	0.0851 (9)	
H14A	0.6001	−0.4060	0.6764	0.128*	
H14B	0.5963	−0.5164	0.5961	0.128*	
H14C	0.5082	−0.4485	0.6293	0.128*	

### Atomic displacement parameters (Å^2^)

**Table:**

	*U*^11^	*U*^22^	*U*^33^	*U*^12^	*U*^13^	*U*^23^
Cl1	0.0740 (5)	0.1134 (6)	0.0590 (4)	0.0144 (4)	−0.0030 (3)	−0.0197 (4)
C10	0.0480 (14)	0.0460 (14)	0.0432 (13)	0.0059 (11)	0.0087 (11)	0.0064 (11)
C11	0.0540 (15)	0.0480 (14)	0.0428 (13)	0.0056 (12)	0.0066 (11)	0.0009 (11)
N2	0.0459 (12)	0.0633 (13)	0.0525 (12)	0.0033 (10)	−0.0018 (10)	0.0017 (10)
O1	0.0624 (11)	0.0998 (14)	0.0659 (12)	0.0237 (10)	0.0059 (9)	−0.0051 (11)
C23	0.0431 (14)	0.0651 (16)	0.0504 (14)	−0.0048 (12)	−0.0039 (11)	0.0000 (13)
N3	0.0499 (12)	0.0747 (14)	0.0498 (12)	0.0013 (11)	−0.0053 (10)	−0.0042 (11)
N1	0.0583 (13)	0.0539 (13)	0.0571 (13)	0.0147 (11)	0.0091 (10)	0.0023 (10)
C9	0.0499 (14)	0.0558 (15)	0.0401 (13)	0.0044 (12)	0.0069 (11)	0.0048 (11)
C3	0.0498 (15)	0.0504 (14)	0.0479 (13)	0.0068 (12)	0.0084 (11)	0.0065 (11)
C12	0.0471 (14)	0.0561 (15)	0.0460 (13)	0.0032 (12)	0.0035 (11)	0.0032 (12)
C4	0.0533 (15)	0.0620 (16)	0.0423 (13)	0.0087 (14)	0.0051 (11)	0.0084 (12)
C2	0.0667 (17)	0.0448 (14)	0.0584 (15)	0.0052 (13)	0.0043 (13)	−0.0011 (12)
C24	0.0496 (15)	0.0677 (17)	0.0495 (14)	−0.0050 (13)	0.0039 (12)	−0.0042 (12)
C18	0.0382 (13)	0.0596 (15)	0.0500 (14)	−0.0034 (11)	−0.0004 (11)	0.0039 (12)
C19	0.0478 (14)	0.0759 (17)	0.0487 (14)	0.0004 (13)	0.0000 (11)	0.0013 (13)
C16	0.0425 (13)	0.0601 (15)	0.0496 (14)	−0.0034 (12)	−0.0005 (11)	0.0004 (12)
C20	0.0449 (15)	0.0738 (17)	0.0562 (16)	0.0009 (13)	0.0029 (12)	−0.0001 (13)
C1	0.0579 (16)	0.0567 (16)	0.0559 (15)	−0.0036 (13)	0.0012 (12)	−0.0006 (12)
C22	0.0516 (15)	0.0836 (19)	0.0533 (15)	0.0021 (14)	−0.0123 (12)	0.0033 (14)
C8	0.0562 (16)	0.0674 (17)	0.0535 (15)	0.0046 (13)	0.0048 (12)	−0.0007 (13)
C17	0.0450 (14)	0.0681 (16)	0.0463 (14)	−0.0043 (13)	−0.0052 (11)	0.0028 (12)
C15	0.0510 (15)	0.0649 (16)	0.0542 (15)	0.0012 (13)	0.0058 (12)	−0.0031 (13)
C6	0.0528 (16)	0.111 (2)	0.0561 (16)	0.0032 (17)	−0.0070 (13)	−0.0003 (17)
C13	0.0713 (17)	0.0633 (16)	0.0612 (16)	0.0199 (14)	0.0127 (14)	−0.0010 (14)
C21	0.0484 (15)	0.0732 (17)	0.0678 (18)	0.0049 (13)	−0.0062 (13)	0.0035 (15)
C5	0.0543 (16)	0.083 (2)	0.0564 (16)	0.0182 (15)	0.0018 (13)	0.0085 (15)
C7	0.0610 (18)	0.083 (2)	0.0625 (17)	0.0000 (16)	−0.0006 (14)	−0.0098 (14)
C25	0.083 (2)	0.117 (3)	0.0600 (18)	0.0211 (18)	0.0132 (16)	−0.0040 (17)
C14	0.117 (3)	0.0603 (18)	0.080 (2)	0.0244 (17)	0.0234 (18)	0.0072 (15)

### Geometric parameters (Å, º)

**Table:**

Cl1—C24	1.752 (2)	C19—C20	1.360 (3)
C10—C11	1.386 (3)	C19—H19	0.9300
C10—C3	1.404 (3)	C16—C17	1.369 (3)
C10—C9	1.449 (3)	C16—C15	1.458 (3)
C11—C12	1.382 (3)	C20—C21	1.403 (3)
C11—H11	0.9300	C1—H1	0.9300
N2—C15	1.269 (3)	C22—C21	1.348 (3)
N2—C12	1.423 (3)	C22—H22	0.9300
O1—C20	1.361 (3)	C8—C7	1.378 (3)
O1—C25	1.425 (3)	C8—H8	0.9300
C23—N3	1.361 (3)	C17—H17	0.9300
C23—C18	1.409 (3)	C15—H15	0.9300
C23—C22	1.413 (3)	C6—C5	1.374 (4)
N3—C24	1.294 (3)	C6—C7	1.382 (4)
N1—C4	1.378 (3)	C6—H6	0.9300
N1—C3	1.380 (3)	C13—C14	1.493 (3)
N1—C13	1.456 (3)	C13—H13A	0.9700
C9—C8	1.389 (3)	C13—H13B	0.9700
C9—C4	1.404 (3)	C21—H21	0.9300
C3—C2	1.382 (3)	C5—H5	0.9300
C12—C1	1.399 (3)	C7—H7	0.9300
C4—C5	1.393 (3)	C25—H25A	0.9600
C2—C1	1.369 (3)	C25—H25B	0.9600
C2—H2	0.9300	C25—H25C	0.9600
C24—C16	1.417 (3)	C14—H14A	0.9600
C18—C17	1.408 (3)	C14—H14B	0.9600
C18—C19	1.409 (3)	C14—H14C	0.9600
			
C11—C10—C3	119.1 (2)	C2—C1—C12	121.5 (2)
C11—C10—C9	134.5 (2)	C2—C1—H1	119.2
C3—C10—C9	106.39 (19)	C12—C1—H1	119.2
C12—C11—C10	119.8 (2)	C21—C22—C23	121.0 (2)
C12—C11—H11	120.1	C21—C22—H22	119.5
C10—C11—H11	120.1	C23—C22—H22	119.5
C15—N2—C12	119.2 (2)	C7—C8—C9	119.0 (2)
C20—O1—C25	117.2 (2)	C7—C8—H8	120.5
N3—C23—C18	122.6 (2)	C9—C8—H8	120.5
N3—C23—C22	119.6 (2)	C16—C17—C18	121.7 (2)
C18—C23—C22	117.8 (2)	C16—C17—H17	119.1
C24—N3—C23	117.3 (2)	C18—C17—H17	119.1
C4—N1—C3	108.95 (19)	N2—C15—C16	121.4 (2)
C4—N1—C13	125.7 (2)	N2—C15—H15	119.3
C3—N1—C13	125.4 (2)	C16—C15—H15	119.3
C8—C9—C4	119.6 (2)	C5—C6—C7	122.5 (3)
C8—C9—C10	133.8 (2)	C5—C6—H6	118.8
C4—C9—C10	106.5 (2)	C7—C6—H6	118.8
N1—C3—C2	129.5 (2)	N1—C13—C14	112.8 (2)
N1—C3—C10	109.1 (2)	N1—C13—H13A	109.0
C2—C3—C10	121.4 (2)	C14—C13—H13A	109.0
C11—C12—C1	119.7 (2)	N1—C13—H13B	109.0
C11—C12—N2	116.8 (2)	C14—C13—H13B	109.0
C1—C12—N2	123.5 (2)	H13A—C13—H13B	107.8
N1—C4—C5	129.5 (2)	C22—C21—C20	120.8 (2)
N1—C4—C9	109.0 (2)	C22—C21—H21	119.6
C5—C4—C9	121.4 (2)	C20—C21—H21	119.6
C1—C2—C3	118.3 (2)	C6—C5—C4	117.1 (2)
C1—C2—H2	120.8	C6—C5—H5	121.5
C3—C2—H2	120.8	C4—C5—H5	121.5
N3—C24—C16	126.5 (2)	C8—C7—C6	120.4 (3)
N3—C24—Cl1	115.38 (18)	C8—C7—H7	119.8
C16—C24—Cl1	118.17 (19)	C6—C7—H7	119.8
C17—C18—C23	116.6 (2)	O1—C25—H25A	109.5
C17—C18—C19	123.2 (2)	O1—C25—H25B	109.5
C23—C18—C19	120.2 (2)	H25A—C25—H25B	109.5
C20—C19—C18	119.9 (2)	O1—C25—H25C	109.5
C20—C19—H19	120.1	H25A—C25—H25C	109.5
C18—C19—H19	120.1	H25B—C25—H25C	109.5
C17—C16—C24	115.2 (2)	C13—C14—H14A	109.5
C17—C16—C15	121.8 (2)	C13—C14—H14B	109.5
C24—C16—C15	122.9 (2)	H14A—C14—H14B	109.5
C19—C20—O1	125.2 (2)	C13—C14—H14C	109.5
C19—C20—C21	120.2 (2)	H14A—C14—H14C	109.5
O1—C20—C21	114.6 (2)	H14B—C14—H14C	109.5
			
C3—C10—C11—C12	−2.3 (3)	C22—C23—C18—C19	2.0 (3)
C9—C10—C11—C12	176.1 (2)	C17—C18—C19—C20	176.0 (2)
C18—C23—N3—C24	−2.7 (3)	C23—C18—C19—C20	−1.4 (4)
C22—C23—N3—C24	177.2 (2)	N3—C24—C16—C17	3.5 (4)
C11—C10—C9—C8	−0.4 (4)	Cl1—C24—C16—C17	−176.18 (17)
C3—C10—C9—C8	178.2 (2)	N3—C24—C16—C15	−173.0 (2)
C11—C10—C9—C4	−178.9 (2)	Cl1—C24—C16—C15	7.3 (3)
C3—C10—C9—C4	−0.4 (2)	C18—C19—C20—O1	−178.3 (2)
C4—N1—C3—C2	180.0 (2)	C18—C19—C20—C21	−0.4 (4)
C13—N1—C3—C2	1.9 (4)	C25—O1—C20—C19	−5.4 (4)
C4—N1—C3—C10	0.0 (2)	C25—O1—C20—C21	176.6 (2)
C13—N1—C3—C10	−178.07 (19)	C3—C2—C1—C12	−0.7 (3)
C11—C10—C3—N1	179.03 (19)	C11—C12—C1—C2	−2.5 (3)
C9—C10—C3—N1	0.2 (2)	N2—C12—C1—C2	176.4 (2)
C11—C10—C3—C2	−1.0 (3)	N3—C23—C22—C21	179.4 (2)
C9—C10—C3—C2	−179.8 (2)	C18—C23—C22—C21	−0.7 (4)
C10—C11—C12—C1	4.0 (3)	C4—C9—C8—C7	1.3 (3)
C10—C11—C12—N2	−174.97 (19)	C10—C9—C8—C7	−177.1 (2)
C15—N2—C12—C11	150.7 (2)	C24—C16—C17—C18	−1.7 (3)
C15—N2—C12—C1	−28.2 (3)	C15—C16—C17—C18	174.9 (2)
C3—N1—C4—C5	−178.0 (2)	C23—C18—C17—C16	−1.9 (3)
C13—N1—C4—C5	0.0 (4)	C19—C18—C17—C16	−179.4 (2)
C3—N1—C4—C9	−0.2 (2)	C12—N2—C15—C16	−177.5 (2)
C13—N1—C4—C9	177.83 (19)	C17—C16—C15—N2	−16.1 (4)
C8—C9—C4—N1	−178.43 (19)	C24—C16—C15—N2	160.2 (2)
C10—C9—C4—N1	0.4 (2)	C4—N1—C13—C14	95.5 (3)
C8—C9—C4—C5	−0.4 (3)	C3—N1—C13—C14	−86.8 (3)
C10—C9—C4—C5	178.4 (2)	C23—C22—C21—C20	−1.1 (4)
N1—C3—C2—C1	−177.5 (2)	C19—C20—C21—C22	1.7 (4)
C10—C3—C2—C1	2.5 (3)	O1—C20—C21—C22	179.8 (2)
C23—N3—C24—C16	−1.4 (4)	C7—C6—C5—C4	1.2 (4)
C23—N3—C24—Cl1	178.33 (17)	N1—C4—C5—C6	176.7 (2)
N3—C23—C18—C17	4.2 (3)	C9—C4—C5—C6	−0.8 (3)
C22—C23—C18—C17	−175.7 (2)	C9—C8—C7—C6	−1.0 (4)
N3—C23—C18—C19	−178.1 (2)	C5—C6—C7—C8	−0.3 (4)

### Hydrogen-bond geometry (Å, º)

Cg1 and Cg2 are the centroids of the C18–C23 and C1–C3/C10–C12 benzene rings, respectively.

**Table:**

*D*—H···*A*	*D*—H	H···*A*	*D*···*A*	*D*—H···*A*
C1—H1···*Cg*1^i^	0.93	2.87	3.718 (3)	152
C7—H7···*Cg*2^ii^	0.93	2.97	3.688 (2)	145

Symmetry codes: (i) −*x*, −*y*, −*z*+1; (ii) −*x*+1, *y*+1/2, −*z*+3/2.
